# When the *JCI* went rogue

**DOI:** 10.1172/jci.insight.202107

**Published:** 2026-01-09

**Authors:** Howard A. Rockman

Ten years ago, we launched *JCI Insight* with both excitement and a fair bit of trepidation. Internally, we affectionately referred to this endeavor as “JCI Rogue,” a recognition of the sense of spirit and adventure that we all felt in embarking on this new path at the *JCI*. We knew the risks. Would the ASCI and the broader scientific community embrace a new journal? Would it truly serve researchers and clinicians? Could it stand alongside *The Journal of Clinical Investigation* — not as a rival, but as a partner in advancing science? And now, a decade later, we can confidently say the answer to all three questions is a resounding yes.

## Why start a new journal?

Early in my tenure as Editor in Chief of the *JCI*, we performed an outcome analysis of all the papers that had been submitted to *JCI* over the previous ten years. Using a variety of search criteria, we identified the fate of approximately 90% of the papers and learned the following: Nearly all had been published, and an overwhelming number were published in very high-quality journals. It became evident we were missing an opportunity to better serve our physician-scientist community. Thus, *JCI Insight* was born from a simple premise: There was outstanding, rigorous biomedical research that deserved a home within the *JCI* family, but the flagship journal could not publish it all.

*JCI Insight* was conceived to meet these needs — a society-owned journal committed to the same principles of excellence and integrity as the *JCI*, but with a focus on the publication of new ideas and emerging discoveries rather than encyclopedic manuscripts. Our mission was not just to create another spin-off journal, but rather a journal that respected authors’ time, that sought a new *insight* into a disease and also discouraged excessive demands on authors during the review process. We were guided by the same principles I wrote about in my *JCI* editorial “Tempus fugit”: enthusiasm, creativity, and integrity (1). These weren’t just abstract ideals; they were the criteria by which our Editorial Board selected manuscripts for publication.

## The first decade

From the first published article to the many that followed, *JCI Insight* has built a track record of publishing research that has impact. In the early days, most papers were transfers from *JCI*. As the years past to the present, now 70% are de novo submissions, whereas 30% are transfers from the *JCI*. Some articles have been highly cited and widely downloaded; and many have seeded additional discoveries. Equally important, the Journal quickly proved to be a vital partner for the ASCI and the broader scientific community. True to its original mission, *JCI Insight* offers authors a fair, rapid, and transparent review process. And it created a financially sustainable model that has helped support the mission of the Society and the *JCI*.

Of course, there were challenges. Balancing speed with rigor is never easy. Maintaining high standards while being author friendly requires an Editorial board of like-minded scientists who believe in the mission. Now ten years on, the journal has not only endured, but it has also thrived.

## What makes *JCI Insight* distinct

As I reflect over the past ten years, I note several elements that have defined *JCI Insight*.

Breadth and scientific purpose. *JCI Insight* has consistently published across the full range of biomedical science — from molecular and cellular biology to translational and clinical studies. Importantly, studies we have published have not needed to present an exhaustively documented story, but rather one that identifies a new pathway, disease process, or potential new therapy.

Commitment to rigor and transparency. From the beginning, the journal has required clear presentation of data and comprehensive and informative figure legends. The goal was to let readers see how conclusions were drawn and not have them struggle to evaluate the data.

Efficiency in peer review. Reviewers were asked to focus on what truly mattered, not to generate extensive lists of new experiments. Authors knew that their work would be judged fairly and quickly, without endless revisions and delays.

Today, the Journal continues to innovate. Research Letters, the Physician-Scientist Development category, themed issues, and author-friendly policies are just the beginning. However, the original mission remains: Publish high-quality, disease-relevant science with speed, fairness, and, of course, insight.

## The next ten years

Looking ahead, the challenges facing scientific publishing are, if anything, even greater than they were a decade ago. Preprints are now routine. Open science practices are expected. Artificial intelligence is reshaping how we analyze, write, and review manuscripts. Readers demand speed and transparency. The current Editorial Board has set out its priorities, and I look forward to seeing *JCI Insight* further evolve while staying true to its core mission: to publish rigorous, meaningful research that advances human health.

## A note of gratitude

No editorial marking a ten-year anniversary would be complete without an expression of gratitude to those whose efforts have made this success possible. *JCI Insight* exists because of its authors, who trusted us with their work; the reviewers, who donated their expertise; the editors, who labored tirelessly behind the scenes; the staff, who made it all work; and to you, the readers, who gave the Journal its purpose.

On behalf of the founding team, I am proud of what we have built together. I look forward to watching *JCI Insight*’s next chapters unfold *—* with the same sense of curiosity, optimism, and, yes, insight that has carried us through the first ten years. And if you ever wonder what makes a journal truly special, remember: It’s not just the science — it’s the soul.

Here’s to the next decade.

## Version 1 01/09/2026

Electronic publication
